# Recurrent severe hypocalcemia following chemotherapy regimen changes in advanced breast cancer: two case reports

**DOI:** 10.1186/s13256-024-04478-3

**Published:** 2024-03-25

**Authors:** Yurina Yanase, Hiroko Bando, Riko Sato, Tomohei Matsuo, Aya Ueda, Mai Okazaki, Sachie Hashimoto, Akiko Iguchi-Manaka, Hisato Hara

**Affiliations:** 1 Department of Breast-Thyroid-Endocrine Surgery, University of Tsukuba Hospital, 2-1-1 Amakubo, Tsukuba-Shi, Ibaraki, 305-8576 Japan; 2 Department of Breast and Endocrine Surgery, Institute of Medicine, University of Tsukuba, 1-1-1 Tennnodai, Tsukuba-Shi, Ibaraki, 305-8575 Japan

**Keywords:** Hypocalcemia, Breast cancer, Advanced breast cancer, Bone metastases, Hungry bone syndrome

## Abstract

**Background:**

As an oncologic emergency related to abnormalities in calcium metabolism, hypercalcemia associated with paraneoplastic syndrome and bone metastases is well known. Meanwhile, the incidence of hypocalcemia is low, except in cases associated with bone-modifying agents used for bone metastases. Hypocalcemia induced by bone-modifying agents typically occurs early after the initial administration, and its incidence can be significantly reduced by preventive administration of calcium and vitamin D3 supplements.

**Case report:**

We report two cases of recurrent severe hypocalcemia occurring during chemotherapy for metastatic breast cancer with multiple bone metastases.

Case 1: A 35-year-old Japanese woman developed metastases in the bone, liver, and ovaries during postoperative endocrine therapy for invasive lobular carcinoma of the breast. She underwent chemotherapy and treatment with denosumab. She experienced recurrent episodes of severe hypocalcemia subsequent to a change in the chemotherapy regimen. Case 2: A 65-year-old Japanese woman encountered multiple bone metastases after postoperative anti-human epidermal growth factor receptor 2 therapy and during endocrine therapy for invasive ductal carcinoma of the breast. She underwent anti-human epidermal growth factor receptor 2 therapy and treatment with denosumab. She experienced recurrent severe hypocalcemia subsequent to a change in the chemotherapy regimen to letrozole + lapatinib, trastuzumab emtansine, and lapatinib + capecitabine.

**Conclusions:**

We observed two cases of recurrent severe hypocalcemia in patients with advanced breast cancer and bone metastases after modifications to their therapy regimens. These cases differed from the typical hypocalcemia induced by bone-modifying agents. It is possible that antitumor drugs affect calcium and bone metabolism associated with bone metastases. While these cases are rare, it is crucial for oncologists to be aware of hypocalcemia not only at the initiation of bone-modifying agents but also throughout the entire antitumor therapy, as hypocalcemia can lead to fatal outcomes.

## Background

Hypocalcemia during cancer drug therapy is known to occur as an adverse event associated with bone-modifying agents (BMAs) in the treatment for bone metastases. Although infrequent, hypocalcemia also occurs as a secondary response to hypoparathyroidism or tumor lysis syndrome.

Denosumab, an anti-receptor activator of nuclear factor κ-Β ligand (RANKL) antibody, is administered to prevent bone resorption and associated symptoms, such as bone pain and pathologic fractures, in bone metastases. Because of the risk that the patient may develop hypocalcemia shortly after the initial administration, preventive calcium and vitamin D3 supplements are often given.

We present two cases of recurrent severe hypocalcemia in advanced breast cancer with bone metastases after modification of the therapy regimens. These cases differed from those with typical denosumab-induced hypocalcemia, and few reports have been published of recurrent hypocalcemia during use of denosumab along with preventive calcium and vitamin D3 supplements.

## Case presentation

### Case 1

The patient was a Japanese woman aged 35 years at diagnosis with no significant past medical history and with a family history of esophageal cancer in her father.

Her medical history revealed that, in year X, the patient was diagnosed with right breast cancer and underwent a total right mastectomy and sentinel lymph node biopsy, which revealed invasive lobular carcinoma, estrogen receptor (ER)-positive (> 90%), progesterone receptor (PgR)- positive (> 90%), human epidermal growth factor receptor 2 (HER-2)-negative, and Ki-67 index < 5%. The pathologic stage was T3N0, stage IIB. She received adjuvant endocrine therapy with leuprorelin and tamoxifen.

**Fig. 1:**
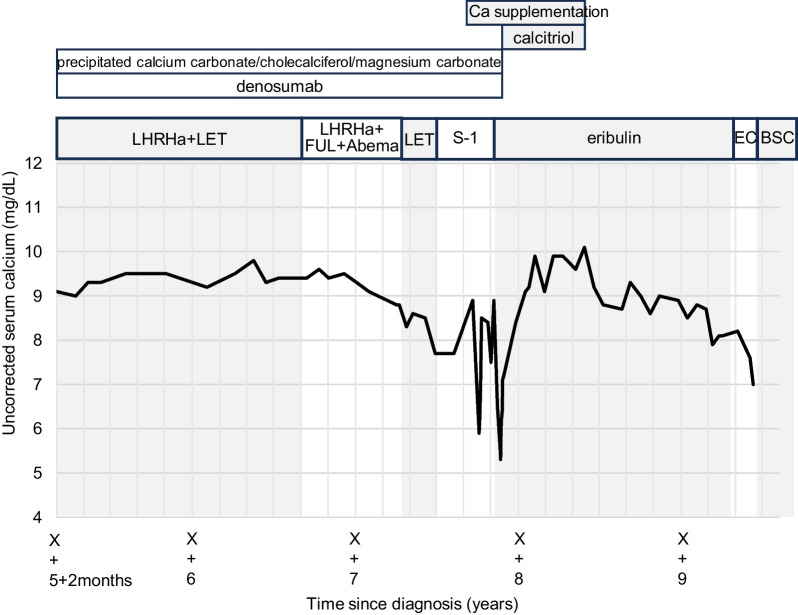
Chemotherapy regimens and trends of serum calcium (uncorrected) in case 1. Severe hypocalcemia recurred on the 99th day of tegafur/gimeracil/oteracil therapy and on the 22nd day of eribulin therapy. Despite the necessity of calcium supplementation, eribulin was continued at a lower dosage

In year X + 5, she had multiple metastatic recurrences in the bone, liver, and ovaries. She began endocrine therapy and treatment with denosumab and precipitated calcium carbonate/cholecalciferol/magnesium carbonate. After third-line endocrine therapy, in year X + 7, she switched to tegafur/gimeracil/oteracil (S-1) as the first-line chemotherapy owing to disease progression. On the 99th day of S-1 therapy, she was urgently hospitalized owing to numbness throughout her body and severe hypocalcemia (serum calcium 5.9 mg/dL). She also exhibited QT prolongation, but no renal dysfunction. The hypocalcemia improved with calcium supplementation (up to 15 g/day). S-1 therapy was switched to eribulin (1.4 mg/mm^2^) in the fourth month. On the 22nd day of eribulin therapy, she was hospitalized again for severe hypocalcemia (serum calcium 5.3 mg/dL). Renal function remained normal. The hypocalcemia improved with discontinuation of denosumab, calcium supplementation (up to 15 g/day), and calcitriol (1.5 μg/day). She resumed eribulin (1.1 mg/mm^2^, subsequently reduced to 0.9 mg/mm^2^) with no recurrence of hypocalcemia. (Fig. 1) In year X + 9, in the 18th month of eribulin treatment, the regimen was switched to epirubicin and cyclophosphamide (EC) therapy. EC therapy was concluded after a single course owing to worsening liver metastases, and she passed away at a palliative care hospital.

### Case 2

The patient was a Japanese woman aged 65 years at diagnosis without a significant past or family medical history.

Her medical history revealed that she was diagnosed with papillary thyroid cancer during an examination for breast cancer and underwent a total thyroidectomy and cervical lymph node dissection. The thyroid cancer was at pathologic stage T3N1aM0, stage III in the 7th edition of UICC. There were no signs of persistent hypoparathyroidism after the total thyroidectomy, and no recurrence was observed until her passing.

In year Y, the patient was diagnosed with invasive ductal carcinoma of the right breast, characterized as ER-positive (> 90%), PgR-positive (> 90%), human epidermal growth factor receptor 2 (HER2)-positive confirmed by fluorescence *in situ* hybridization (FISH), and the Ki-67 index was 16.3%. The clinical stage was T3N1M0, stage IIIA. After neoadjuvant chemotherapy, she underwent a right partial mastectomy and axillary lymph node dissection, achieving a complete response. She received adjuvant therapy with anastrozole and trastuzumab.

**Fig. 2:**
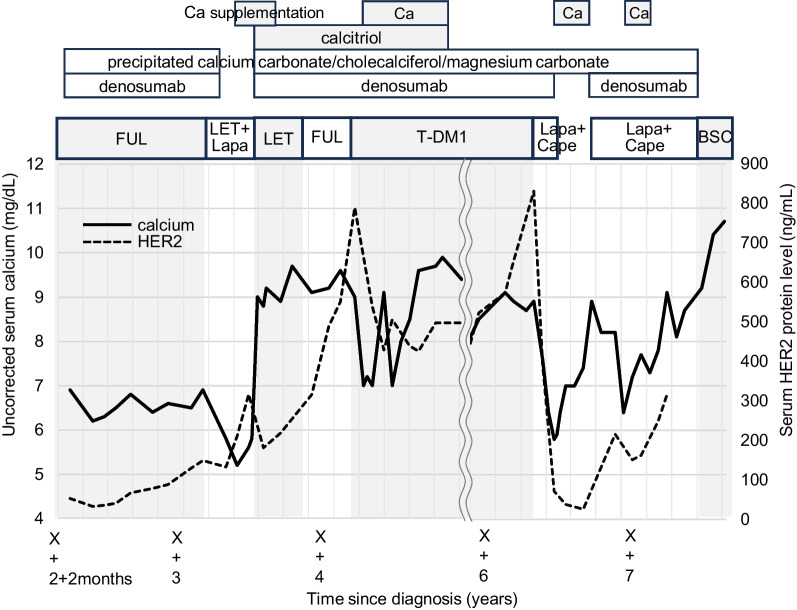
Chemotherapy regimens and trends of serum calcium (uncorrected) and serum human epidermal growth factor receptor 2 protein in case 2. Severe hypocalcemia recurred on the 56th day of letrozole therapy, on the 42nd day of trastuzumab emtansine therapy, on the 35th day of lapatinib and capecitabine therapy, and on the 21st day of resumed lapatinib and capecitabine therapy. The serum human epidermal growth factor receptor 2 protein levels decreased after the initiation of the trastuzumab emtansine or lapatinib and capecitabine therapies, showing a tendency to correlate with the trends in serum calcium levels

In year Y + 2, she developed multiple bone metastases and received fulvestrant, denosumab, and precipitated calcium carbonate/cholecalciferol/magnesium carbonate. In year Y + 3, the regimen was switched to letrozole and lapatinib therapy owing to bone metastases progression. On the 56th day of letrozole and lapatinib therapy, she exhibited severe hypocalcemia (serum calcium 5.2 mg/dL) and QT prolongation during a routine check-up. She only complained of dry mouth and showed no signs of tetany. The hypocalcemia improved with calcium supplementation (up to 9 g/day) and calcitriol (1.5 μg/day). She had a positive response to letrozole monotherapy for 4 months followed by fulvestrant for 3 months. The regimen was switched to trastuzumab emtansine (T-DM1) owing to progression of the bone metastases. On the 42nd day of T-DM1 therapy, she exhibited hypocalcemia (serum calcium 7.0 mg/dL) and a decrease in serum HER2 protein levels (from 790 to 428 ng/mL). After improvement of hypocalcemia, she resumed T-DM1 therapy and had a positive response for 26 months. The regimen was switched to lapatinib and capecitabine owing to bone metastases progression. On the 35th day, she developed hypocalcemia (serum calcium 5.8 mg/dL) along with a decrease in serum HER2 protein levels (from 830 to 24.9 ng/mL). The lapatinib and capecitabine therapy was suspended for 3 months. On the 21st day after resumption of lapatinib and capecitabine therapy, she again experienced hypocalcemia (serum calcium 6.4 mg/dL). Throughout the course, renal function remained normal (Fig. 2). In year Y + 7, on the 9th month of lapatinib and capecitabine therapy, her condition rapidly deteriorated because of liver metastases and she passed away at the hospital.

## Discussion

### Clinical course of hypocalcemia in the two cases

We report two cases of recurrent severe hypocalcemia in breast cancer. Both patients had multiple bone metastases; case 1 was the luminal type, and case 2 was the luminal HER2 positive type. The patients did not experience hypocalcemia shortly after the first denosumab administration, but did so more than a year later despite use of preventive calcium and vitamin D supplements. This differed from the typical denosumab-induced hypocalcemia. There were no other causes of hypocalcemia, such as renal dysfunction, hypoparathyroidism, calcium loss from the kidneys, or hypomagnesemia. Calcium abnormalities owing to drug interactions were also unlikely, given case 1 involved sodium ferrous citrate and magnesium oxide, and case 2 included levothyroxine sodium hydrate, lansoprazole, and acetaminophen.

Additionally, severe and acute hypocalcemia recurred after the regimen changes. In case 1, the calcium levels remained stable until the third endocrine therapy, but hypocalcemia recurred after S-1 and eribulin therapy (Fig. 1). In case 2, the calcium levels remained stable during endocrine therapy, but hypocalcemia recurred after anti-HER2 therapy. The serum HER2 protein levels tended to decrease along with the serum calcium levels (Fig. 2), suggesting that the development of hypocalcemia could be associated with effective drug therapies. At 2–4 months after the treatment changes, the serum calcium level became stable and the amount of required calcium supplementation decreased.

### Calcium abnormalities associated with cancer

Hypercalcemia associated with cancer is well known as an oncologic emergency, whereas hypocalcemia is less common. The primary causes of hypercalcemia include humoral hypercalcemia of malignancy (HHM) and osteolytic bone metastases. HHM involves excessive secretion of parathyroid hormone-related peptide (PTHrP) and is frequently observed in squamous cell carcinoma of the lungs, breast cancer, ovarian cancer, and renal carcinoma. Hypercalcemia due to osteolytic bone metastases is commonly seen in patients with multiple myeloma and solid organ tumors, such as breast cancer [1, 2].

Hypocalcemia is usually associated with adverse effects of BMAs, such as denosumab and bisphosphonates, and also of hypoparathyroidism, hypomagnesemia, vitamin D deficiency, and intestinal malabsorption [3]. Hypocalcemia occurs in 5.5–13% of patients receiving denosumab [4–6] within 6 months (often within 2 weeks) of initiation [5–8]. This happens because bone-forming osteoblasts become dominant over bone-resorbing osteoclasts. Osteoclasts are suppressed within weeks, while osteoblasts persist for several months after denosumab administration. The risk factors for denosumab-induced hypocalcemia include prostate cancer, small cell lung cancer, multiple osteoblastic bone metastases, renal dysfunction, and elevated bone-specific alkaline phosphatase levels [9, 10]. Preventive intake of calcium and vitamin D3 agents can reportedly reduce the incidence of hypocalcemia by 40% [10].

### Relationship between hypocalcemia and antitumor drug therapy

Reports on chemotherapy-induced hypocalcemia in patients not using BMAs are limited to only one case of Hodgkin lymphoma [11].

Hungry bone syndrome is a condition of persistent hypocalcemia owing to significant absorption of calcium into the bones. It occurs in patients with severe hyperparathyroidism after surgical treatments, such as total parathyroidectomy or kidney transplantation [12, 13]. In prostate cancer, osteoblastic bone metastases can cause hypocalcemia similar to hungry bone syndrome as a result of excessive calcium uptake in the bone [14, 15].

Breast cancer bone metastases are typically osteolytic or mixed type [16]. The two patients here had mainly multiple osteolytic bone metastases and were treated with denosumab. They became resistant to previous therapies as the disease progressed, and underwent another chemotherapy and anti-HER2 therapy. We consider that this led to a rapid shift in bone metabolism from osteolytic to osteoblastic, causing significant calcium absorption into the bone. This contributed to severe hypocalcemia similar to hungry bone syndrome.

### Symptoms and management of hypocalcemia

Initial hypocalcemia is often asymptomatic but can sometimes exhibit the Chvostek and Trousseau signs, tetany symptoms, electrocardiogram (ECG) abnormalities, such as prolonged QTc and ST intervals, arrhythmias, seizures, and even fatality [15, 17]. Case 1 exhibited tetany symptoms, whereas case 2 did not. In both cases, hypocalcemia improved within a few days with calcium supplements (calcium lactate 3–15 g/day, calcium gluconate infusion 3.9–23.4 mEq/day) and vitamin D3 supplements (calcitriol 1.5 μg/day). Furthermore, the chemotherapy treatment could be continued by discontinuing denosumab, interrupting or reducing the chemotherapy doses, and giving calcium supplements. Regular calcium level monitoring through blood tests was considered important during antitumor treatment.

## Conclusion

We report herein two cases of metastatic breast cancer with recurrent severe hypocalcemia subsequent to treatment regimen changes. Although hypocalcemia associated with cancer is uncommon, it is essential to recognize that hypocalcemia can occur during antitumor therapy, especially in patients with multiple bone metastases, regardless of the histologic type or subtype of the cancer. We emphasize the need for continuous calcium level monitoring not only at the initiation of treatment with BMAs but also throughout the entire treatment. While hypocalcemia can become fatal, it can be improved with appropriate treatment.

## Data Availability

The data used and analyzed in this study are available from the corresponding author upon reasonable request.

## Abbreviations

BMAs: Bone-modifying agents
HER2: Human epidermal growth factor receptor 2
ER: Estrogen receptor
PgR: Progesterone receptor
S-1: Tegafur/gimeracil/oteracil
EC: Epirubicin and cyclophosphamide
BCS: Best supportive care.
FISH: Fluorescence in situ hybridization
T-DM1: Trastuzumab emtansine
Ca: Calcium
LHRHa: Luteinizing hormone-releasing hormone agonist
LET: Letrozole
FUL: Fulvestrant
Abema: Abemaciclib
Lapa: Lapatinib
Cape: Capecitabine
